# Children’s Health: Methylmercury and Children’s Heart Function

**Published:** 2004-11

**Authors:** John Tibbetts

Pregnant women who consume significant amounts of seafood may have a new reason to take precautions against methylmercury, the most hazardous form of mercury: a recent study suggests that when expectant women consume fish containing high levels of the toxicant, their children’s future cardiovascular health may be jeopardized.

Fish and shellfish are the main sources of exposure to methylmercury for most Americans. Methylmercury tends to accumulate the most in large predatory species such as yellowfin tuna, shark, swordfish, and marlin. Other commonly eaten species can accumulate intermediate levels of methylmercury. Fish with the lowest mercury content include cod, flounder, salmon, herring, and smaller tuna species that Americans buy canned.

In 1986, researchers led by Harvard environmental epidemiologist Philippe Grandjean and Faroese Hospital System chief physician Pal Weihe began a long-term study of mothers in the Faroe Islands and their children. The Faroese are among the world’s leading seafood consumers per capita, with the average islander eating 2.4 ounces of fish per day. This diet exposes them to increased amounts of methylmercury.

Over a 21-month period, the researchers gathered a cohort of 1,022 women giving birth in the Faroe Islands. They tested mercury concentrations in the children by analyzing cord blood samples at birth and blood and hair samples taken at ages 7 and 14 years. They also measured the mercury in each woman’s hair by taking a sample at the time of parturition.

In one of the latest papers to come from this study, published in the February 2004 issue of the *Journal of Pediatrics*, Grandjean and his colleagues report that mercury which passed from mother to child *in utero*, first measured in cord blood, produced long-lasting harm to the child’s neurologic mechanism that regulates heart function, as measured by heart rate variability. At higher mercury exposures, children were less capable of maintaining normal heart rate variability, which is a risk factor for development of heart disease. The decrease in heart rate variability at increasing mercury exposures was the steepest in the low range of mercury exposures, around the U.S. Environmental Protection Agency exposure limit. When the exposures increased above twice that limit, the effect was not as clear.

Very little is known about the impact of heart rate variability in children, except that children with congenital heart disease also have lower heart rate variability. Grandjean says, “The mercury-associated changes in the Faroe Islands study persisted at least to age fourteen, and it’s possible that they are permanent. In adults, decreased heart rate variability is a known risk factor for heart disease mortality.”

Alan Stern, an adjunct associate professor of public health at the University of Medicine and Dentistry of New Jersey, points out that because this effect is likely the result of developmental changes in the children’s neurologic systems, it may be a sentinel for other neurophysiological disturbances. The developing brain is particularly vulnerable to methylmercury, and brain damage incurred during development is likely to be permanent.

However, Gary Myers, a pediatric neurologist who studies mercury exposure at the University of Rochester in New York, says that the Faroese are unusual in their diet of whale meat, which is especially high in concentrations of mercury and other toxicants. Therefore, he says, this study cannot be generalized to the United States and other countries with populations that do not consume whale meat.

But many Faroese do not eat whale, says Grandjean, and its availability varies seasonally and among communities. He says mercury associations found at low-exposure levels are more likely to be related to other kinds of seafood with high mercury concentrations. Still, he cautions that scientific conclusions should not be based on a single study. Moreover, consumers should not be scared away from eating seafood, but should instead be wary of fish with elevated mercury concentrations, particularly large predatory species.

## Figures and Tables

**Figure:**
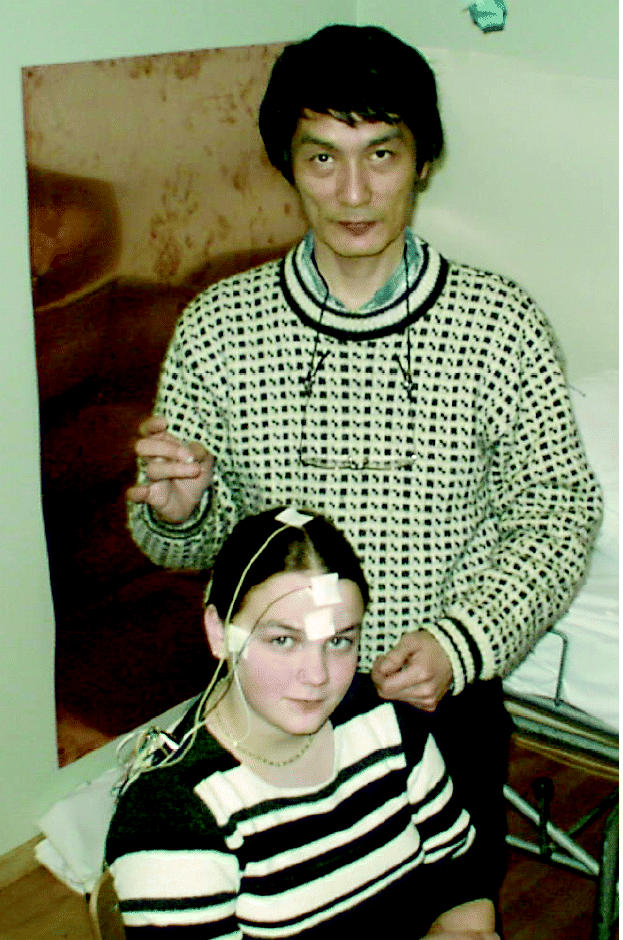

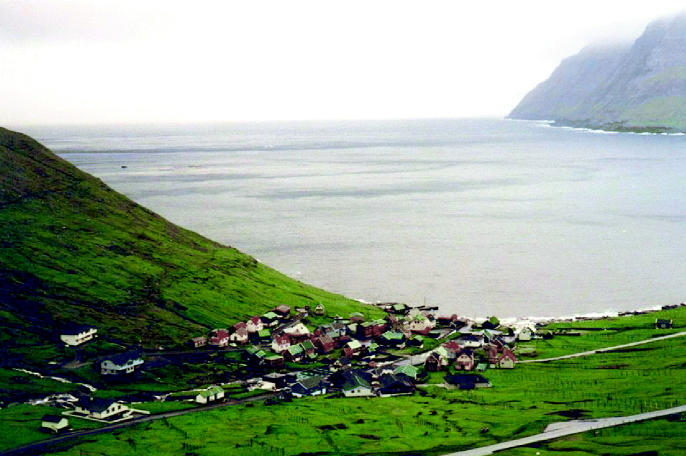
**Skipping a beat.** New data from studies of children in the Faroe Islands exposed *in utero* to methylmercury show long-lasting effects on the neurological mechanism that controls heart rate variability.

